# Altered hypothalamic functional connectivity in post-traumatic headache after mild traumatic brain injury

**DOI:** 10.1186/s10194-020-01164-9

**Published:** 2020-07-28

**Authors:** Liyan Lu, Fengfang Li, Peng Wang, Huiyou Chen, Yu-Chen Chen, Xindao Yin

**Affiliations:** Department of Radiology, Nanjing First Hospital, Nanjing Medical University, No.68, Changle Road, Nanjing, 210006 China

**Keywords:** Functional connectivity, Hypothalamus, Post-traumatic headache, Mild traumatic brain injury

## Abstract

**Background:**

Post-traumatic headache (PTH) is one of the most frequent symptoms following mild traumatic brain injury (mTBI). Neuroimaging studies implicate hypothalamic function connectivity (FC) disruption as an important factor in pain disorders. However, it is unknown whether there are alterations in the hypothalamus-based resting state FC within PTH following mTBI at the acute stage and its relationship with headache symptom measurement.

**Methods:**

Forty-four mTBI patients with PTH, 27 mTBI patients without PTH and 43 healthy controls who were well matched for age, gender, and years of education were enrolled in this study. All participants underwent resting-state functional magnetic resonance imaging (fMRI) scanning as well as headache symptom measurement and cognitive assessment. Hypothalamic resting state networks were characterized by using a standard seed-based whole-brain correlation method. The bilateral hypothalamic FC was compared among the three groups. Furthermore, the correlations between hypothalamic resting state networks and headache frequency, headache intensity and MoCA scores was investigated in mTBI patients with PTH using Pearson rank correlation.

**Results:**

Compared with mTBI patients without PTH, mTBI patients with PTH at the acute stage presented significantly decreased left hypothalamus-based FC with the right middle frontal gyrus (MFG) and right medial superior frontal gyrus (mSFG), and significantly decreased right hypothalamus-based FC with the right MFG. Decreased FC of the right MFG was significantly positively associated with headache frequency and headache intensity (*r* = 0.339, *p* = 0.024; *r* = 0.408, *p* = 0.006, respectively). Decreased FC of the right mSFG was significantly positively associated with headache frequency and headache intensity (*r* = 0.740, *p* < 0.0001; *r* = 0.655, *p* < 0.0001, respectively).

**Conclusion:**

Our data provided evidence of disrupted hypothalamic FC in patients with acute mTBI with PTH, while abnormal FC significantly correlated with headache symptom measurement. Taken together, these changes may play an essential role in the neuropathological mechanism of mTBI patients with PTH.

## Background

Traumatic brain injury (TBI) is a significant worldwide health problem with major effects on both morbidity and mortality [1]. Among all traumatic brain injuries, mild traumatic brain injury (mTBI) is highly prevalent [2]. Following mTBI, patients frequently suffer lifelong disabilities, including post-traumatic headache (PTH), depression, insomnia, dizziness and hypomnesia. PTH is the most common and persistent symptom, and almost 70% of individuals with mTBI report pain-related problems and complaints. According to the International Classification of Headache Disorders Third Edition (ICHD-3), PTH is considered as an “acute” headache disorder if it develops within 3 months after the injury and as a “persistent” condition if it continues for longer than 3 months [3]. Persistent post traumatic injury (PPTH) may sometimes be underestimated because many patients never go to the hospital to obtain headache medication, which ultimately leads to long-lasting disability and imposes major burdens on society. However, it is still challenging for us to identify patients with mTBI with PTH, since the conventional computed tomography (CT) and magnetic resonance imaging (MRI)) cannot identify brain abnormalities [4]. Therefore, the underlying pathophysiologic mechanisms of PTH following mTBI are poorly understood.

The main phenotype of PTH in patients with mTBI is consistent with that of migraine [5, 6], and PTH commonly has characteristics similar to migraine. Thus, there are shared underlying neurochemical mechanisms between migraine and PTH [7]. Several studies have shown that various brain structures, such as the brainstem, central dopaminergic system and hypothalamus, are involved in the pathophysiology of migraine [8–10]. In particular, recently, the hypothalamus has been suggested to play an important role in the generation of migraine attacks and the sustainment of migraine pain [8]. Furthermore, in migraine, the hypothalamus has been reported to be involved not only in different stages of the migraine cycle (interictal, preictal and ictal) and the pathophysiology of migraine chronification, but also in clinical features of migraine, including yawning, tiredness and mood changes [8]. How hypothalamic changes might result in migraine is more intriguing. Neuroimaging studies revealed structural and functional alterations of the hypothalamus within migraine. One recent study showed that the volume of the hypothalamus was significantly decreased in migraine patients, compared with controls and that the volume of the hypothalamus was negatively correlated with headache frequency [10]. Additionally, several studies using functional MRI (fMRI) have indicated that migraine patients had both increased hypothalamic functional connectivity (FC) with a number of brain regions, including the frontal, parietal, temporal, subcortical, brainstem and cerebellum regions, and decreased hypothalamic FC with frontal and occipital regions [9, 11]. Given some overlapping pathophysiological features underlying migraine and PTH, the hypothalamus is presumed to be an important biomarker for the diagnosis and treatment of PTH. Morphological differences (including multifocal lower gray matter, cortical thinning and volume loss and functional alterations regarding pain processing) have been shown in patients with PTH after mTBI. Dumkrieger et al. reported that headache frequency was significantly correlated with static FC of the right posterior insula with the left hypothalamus in PTH [12]. However, to date, no study directly focusing on the hypothalamic FC alterations has been conducted in PTH following mTBI.

Thus, the present study aimed to investigate the FC between the hypothalamus and other brain regions in mTBI patients with PTH and further explore the association between any functional brain abnormality and various clinical features of PTH. By doing so, we utimately aimed to detect the possible pathophysiologic mechanisms of the hypothalamus in PTH following acute mTBI.

## Methods

### Subjects

This study was approved by the Institutional Review Board of Nanjing Medical University. All subjects provided written informed consent before undergoing MRI.

Between December 2017 and January 2019, patients with a diagnosis of mTBI were prospectively enrolled in this study. mTBI was defined based on the American Congress of Rehabitation Medicine [13]. Inclusion criteria were as follows: (a) patients aged 18 or older; (b) loss of consciousness<30 min; (c) Glasgow Coma Score (GCS) of 13–15; and (d)post-traumatic amnesia<24 h. Exclusion criteria were as follows: (a) previous head injury; (b) history of pre-existing neurological or psychiatric disease; (c) history of illicit drug use or substance abuse; (d) dental appliances that might distort the functional MR images; (e) left-handedness; and (f) history of migraine or any other headache prior to injury. The healthy control participants were recruited through local advertisements who met the same exclusion criteria applied to the patient group.

### Headache symptom measurement

After a duration of 12 months, two experienced headache specialists confirmed all headache diagnoses using International Classification of Headache Disorders 3rd edition, beta version (ICHD-3 beta) diagnostic criteria [3]. Participants with PTH provided detailed information about their headaches including predominant localization, headache frequency (days/months) and headache intensity. Participants completed the Visual Analog Scale (VAS) [14], a numeric rating scale ranging from 0 to 10 where 0 represents no pain and 10 represents the most severe pain imaginable to report their headache intensity.

### Cognitive evaluation

Given the emergency care setting, it was not feasible to perform a full battery of cognitive assessments. Therefore, a short instrument called the Montreal Cognitive Assessment (MoCA) was used to assess the patients’ neurocognitive status [15]. The MoCA is a sensitive cognitive screening test following mTBI, and it only requires limited training to administer. The MoCA has been extensively used in patient with pain disorders and in the study of Santangelo et al. [16]. This test is administered in approximately 10 min and is scored on a maximum of 30 points. Scores greater than 26 were regarded as normal values, with lower scores indicating greater cognitive deficit. All participants completed the MoCA test within 48 h of MRI examination.

### Imaging acquisition

A 3.0 T magnetic resonance imaging scanner (Ingenia, Philips Medical Systems, Netherlands) with an 8-channel head coil was used for this study. Functional images were obtained axially using a gradient echo-planar imaging sequence as follows: repetition time (TR) = 2000 ms; echo time (TE) = 30 ms; slices = 36; thickness = 4 mm; gap = 0 mm; field of view (FOV) = 240 mm × 240 mm; acquisition matrix = 64 × 64; and flip angle (FA) = 90°. The fMRI sequence took 8 min and 8 s. The three-dimensional turbo fast-echo (3D-TFE) T1WI sequence had high resolution: TR = 8.1 ms; TE = 3.7 ms; slices = 170; thickness = 1 mm; gap = 0 mm; FA = 8°; acquisition matrix = 256 × 256; and FOV = 256 mm × 256 mm; For fluid-attenuated inversion recovery (FLAIR), the specifications were as follows: TR = 7000 ms; TE = 120 ms; slices = 18; slice thickness = 6 mm; gap = 1.3 mm; FA = 110°; and voxel size = 0.65 × 0.95 × 6 mm^3^. The specifications for susceptibility weighted imaging (SWI) were as follows: TR = 22 mm; TE = 34 ms; FA = 20; matrix = 276 × 319; slice thickness = 1 mm; and FOV = 220 mm × 220 mm. SWI used a 3D gradient echo (GRE) sequence. FLAIR and SWI were used to investigate the presence of traumatic lesions. Traumatic lesions could be shown by high signals on FLAIR and low signals on SWI.

### Image processing and analysis

Functional image analyses were preprocessed with the toolbox Data Processing Assistant for Resting-State fMRI programs based on Statistical Parametric Mapping (SPM8, http://www.fil.ion.ucl.ac.uk/spm) and the resting-state fMRI data analysis toolkit (REST, http://www.restfmri.net). The first 10 volumes were discarded and the remaining 230 consecutive volumes were used for data analysis. Afterwards, slice-timing adjustment and realignment for head motion correction were performed. Any participant who had a head motion greater than 3.0 mm or a rotation in the x, y, or z directions higher than 3.0° were excluded. Data were spatially normalized to the Montreal Neurological Institute (MNI) template (resampling voxel size = 3 × 3 × 3 mm^3^) and smoothed with a Gaussian kernel of 6 mm full width at half maximum (FWHM) to increase the signal-to-noise ratio.

To examine the FC patterns for the hypothalamus, the seed ROIs of the left and right hypothalamus were generated using the WFU PickAtlas software. The mean time series of each ROI was acquired for the reference time course. Then, Pearson’s correlation coefficients were computed between the mean signal change of each ROI and the time series of each voxel. Finally, a Fisher’s z-transform was applied to improve the normality of the correlation coefficients. Differences in FC of the bilateral hypothalamus were compared between groups (mTBI vs. healthy controls; mTBI+PTH vs. mTBI-PTH; mTBI+PTH vs. healthy controls; mTBI-PTH vs. healthy controls) using one-way analysis of variance (ANOVA) and subsequent t-tests at a threshold of *p* < 0.001 with multiple comparisons correction using the AlphaSim program (http://afni.nih.gov/afni/docpdf/AlphaSim.pdf) determined by Monte Carlo simulation (single voxel *p* value = 0.001, a minimum cluster size of 13, within a GM mask corresponding to the AAL atlas) and controlling for age and gender.

### Statistical analyses

Differences in demographic data among mTBI patients with PTH, mTBI patients without PTH and healthy controls were analyzed using one-way ANOVA. Furthermore, a post hoc test (t-test for means and χ2-test for proportions) was performed between mTBI patients with PTH and mTBI patients without PTH. *P* < 0.05 was considered to be statistically significant.

To investigate the abnormal hypothalamic FC between mTBI patients with PTH, mTBI patients without PTH and healthy controls, one-way ANOVA and subsequent t-tests for each seed region were estimated. Then, surface-based cluster-wise correction for multiple comparisons was performed at the significance threshold of *p* < 0.001 and the cluster-size threshold of 13 mm^2^, which was determined by Monte Carlo simulation (single voxel *p* value = 0.001, a minimum cluster size of 13, within a GM mask corresponding to the AAL atlas). The correlation between fMRI data and headache characteristics (headache frequency and headache intensity) and between MoCA scores in participant cohorts was investigated by using Pearson’s correlation analyses. We considered statistically significant Pearson’s correlations with *p* values lower than 0.05.

## Results

After follow-up evaluation, 71 patients with mTBI and 43 healthy controls were enrolled in this study. All the participants were divided into three groups: mTBI + PTH (patients with PTH after mTBI, *n* = 44); mTBI – PTH (patients without PTH after mTBI, *n* = 27); and healthy controls (healthy volunteers without any headache, *n* = 43). Table 1 is a summary of the basic demographic characteristics of the mTBI + PTH group, mTBI – PTH group and healthy control group. These three groups are well-matched and did not show any significant differences in age (*p* = 0.624), gender (*p* = 1.000), years of education (*p* = 0.569) or MoCA (*p* = 0.300). No visible traumatic lesions were seen on conventional imaging such as FLAIR or SWI.

**Table 1:** Clinical and demographic data of the study population

**Characteristics**	**mTBI + PTH** **(*n*** **= 44)**	**mTBI-PTH** **(*n*** **= 27)**	**HCs** **(*n*** **= 43)**	**Group comparison** ***P*values**
**Age (y)**	40.9 ± 10.9	40.5 ± 10.6	43.5 ± 10.8	0.624
**Gender (male/female)**	17/27	14/13	17/26	1.000
**Education (y)**	11.8 ± 3.5	11.1 ± 4.0	12.7 ± 2.9	0.569
**GCS Score**	15	15	15	
**MoCA**	24.5 ± 2.6	24.4 ± 3.2	25.7 ± 2.5	0.300
**Headache characteristics**				
**Predominant side**				
Left	17	–	–	
Right	9	–	–	
Bilateral	10	–	–	
Unilateral	8	–	–	
**Onset**	12.0 ± 7.9	–	–	
**Headache frequency**	12.0 ± 7.4	–	–	
**Headache intensity**	4.7 ± 2.0	–	–	

Data are the mean ± standard deviation; *mTBI* mild traumatic brain injury; *PTH* post-traumatic headache; *HCs* healthy controls; *GCS* Glasgow Coma Scale; *MoCA* Montreal Cognitive Assessment

### Whole patients with mTBI vs healthy controls

**Fig. 1:**
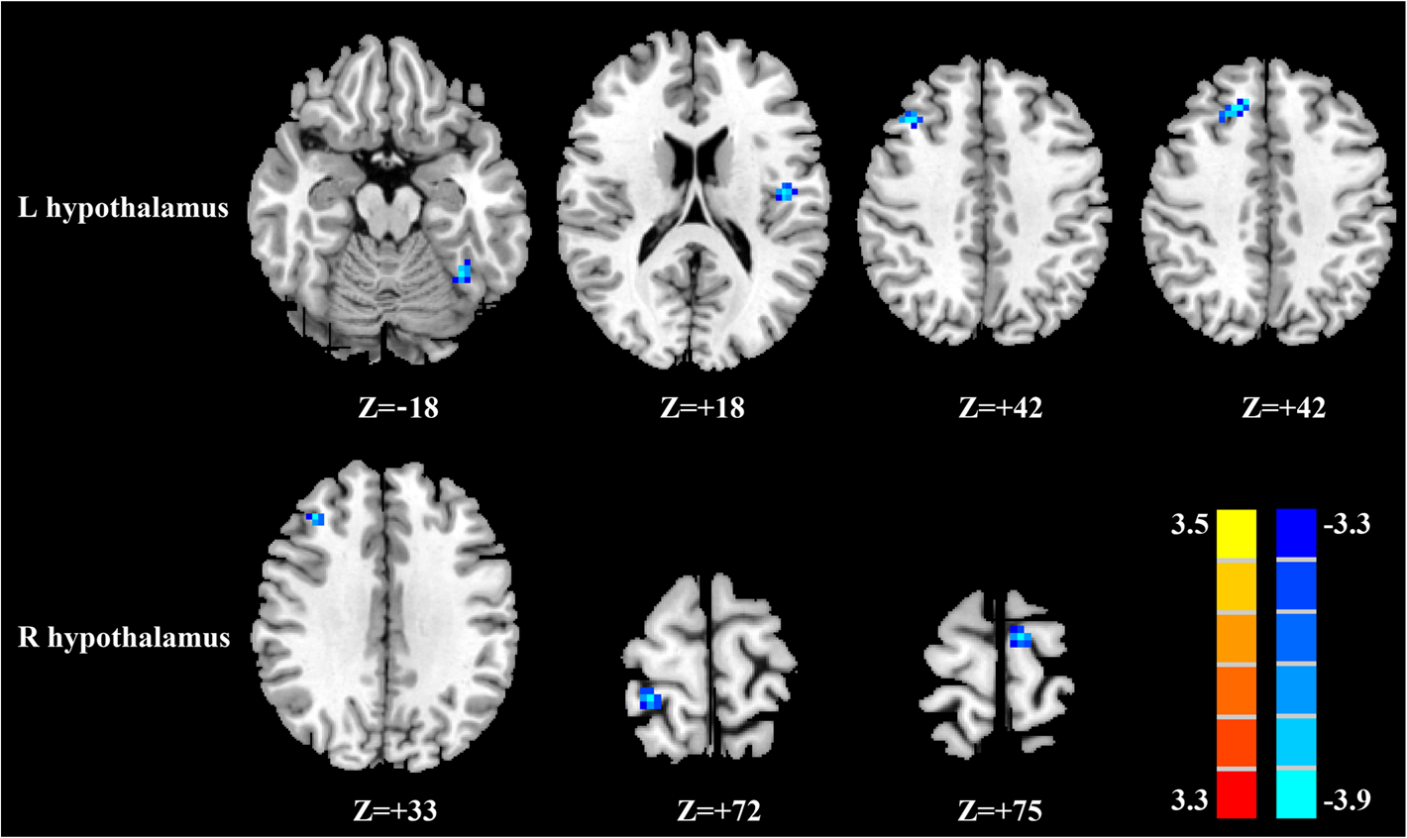
Significant differences in the hypothalamic functional connectivity between patients with mTBI and healthy controls. From the left hypothalamus to the whole brain regions (top row); from the right hypothalamus to the whole brain regions (bottom row). Thresholds were set at a corrected *p* < 0.001, determined by Monte Carlo simulation

Compared with healthy controls, all whole patients with mTBI demonstrated significantly reduced FC between the left hypothalamus and left fusiform gyrus, left Rolandic operculum, and right middle frontal gyrus (MFG) as well as reduced FC between the left hypothalamus and right medial superior frontal gyrus (mSFG) (Fig. 1, Table 2). All whole patients with mTBI exhibited significantly decreased FC between the right hypothalamus and right MFG and right postcentral gyrus as well as decreased FC between the right hypothalamus and left supplementary motor area relative to healthy controls (Fig. 1, Table 2).

**Table 2:** Significant brain regions showing altered hypothalamus functional connectivity in mTBI patients compared with healthy controls

**Brain region**	**BA**	**Peak MNI coordinates** **x,y,z (mm)**	**t value**	**Voxels**
**L hypothalamus**				
L fusiform gyrus	37	−36,-54,-18	−4.1003	17
L Rolandic operculum	45	−45,-12,18	−4.1043	48
R middle frontal gyrus	9	36,24,42	−3.8419	36
R medial superior frontal gyrus	10	12,33,42	−3.7560	27
**R hypothalamus**				
R middle frontal gyrus	9	36,33,33	−4.6247	17
R postcentral gyrus	2	24,-39,72	−4.6080	23
L supplementary motor area	6	−9,-12,75	−4.4478	18

A corrected threshold of *p*<0.001 determined by Monte Carlo simulation was taken as measuring that there was significant difference between groups. *BA* Brodmann area; *MNI* Montreal Neurological Institute; *L* left; *R* right; *mTBI* mild traumatic brain injury

### mTBI patients with PTH vs healthy controls; mTBI patients without PTH

**Fig. 2:**
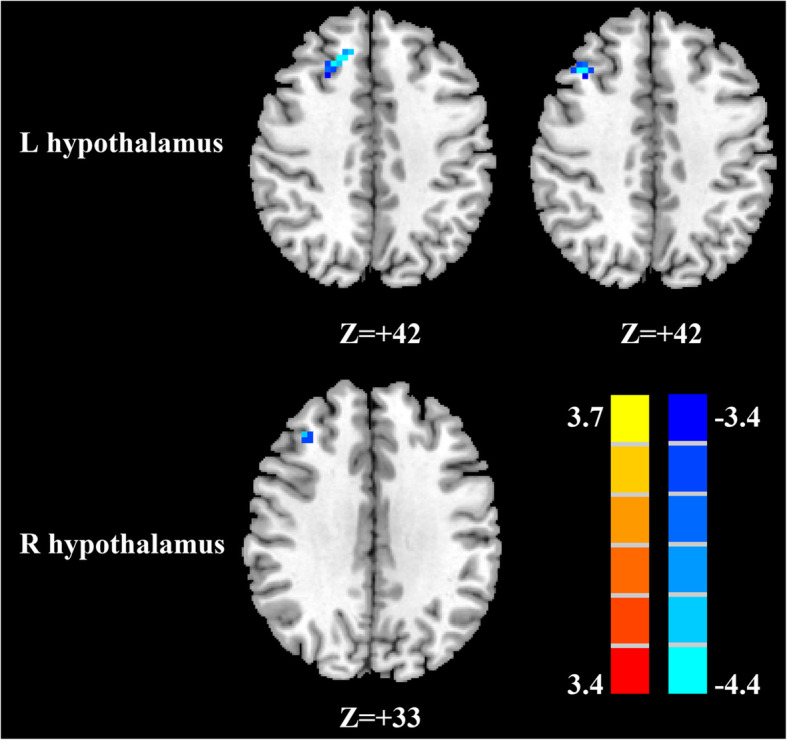
Significant differences in the hypothalamic functional connectivity between patients with mTBI with PTH and patients with mTBI without PTH. (A) From the left hypothalamus to the whole brain regions (top row); from the right hypothalamus to the whole brain regions (bottom row). Thresholds were set at a corrected *p* < 0.001, determined by Monte Carlo simulation

Compared with mTBI patients without PTH, mTBI patients with PTH showed a significant FC reduction between the left hypothalamus and right mSFG and right MFG as well as decreased FC between the right hypothalamus and right MFG (Fig. 2, Table 3).

**Table 3:** Significant brain regions showing altered hypothalamus functional connectivity in mTBI patients with PTH compared with mTBI patients without PTH

**Brain region**	**BA**	**Peak MNI coordinates x,y,z (mm)**	**t value**	**Voxels**
**L hypothalamus**				
R superior medial frontal gyrus	10	18,30,42	−3.8630	34
R middle frontal gyrus	9	39,24,42	−4.0160	22
**R hypothalamus**				
R middle frontal gyrus	9	36,33,33	−4.3566	15

A corrected threshold of *p*<0.001 determined by Monte Carlo simulation was taken as measuring that there was significant difference between groups. *BA* Brodmann area; *MNI* Montreal Neurological Institute; *L* left; *R* right; *mTBI* mild traumatic brain injury; *PTH* post traumatic headache

### mTBI patients with PTH vs healthy controls; mTBI patients without PTH vs healthy controls

Compared with healthy controls, mTBI patients without PTH and mTBI patients without PTH showed no significant difference, respectively.

### Correlation between the hypothalamic FC of identified regions and headache measurements

**Fig. 3:**
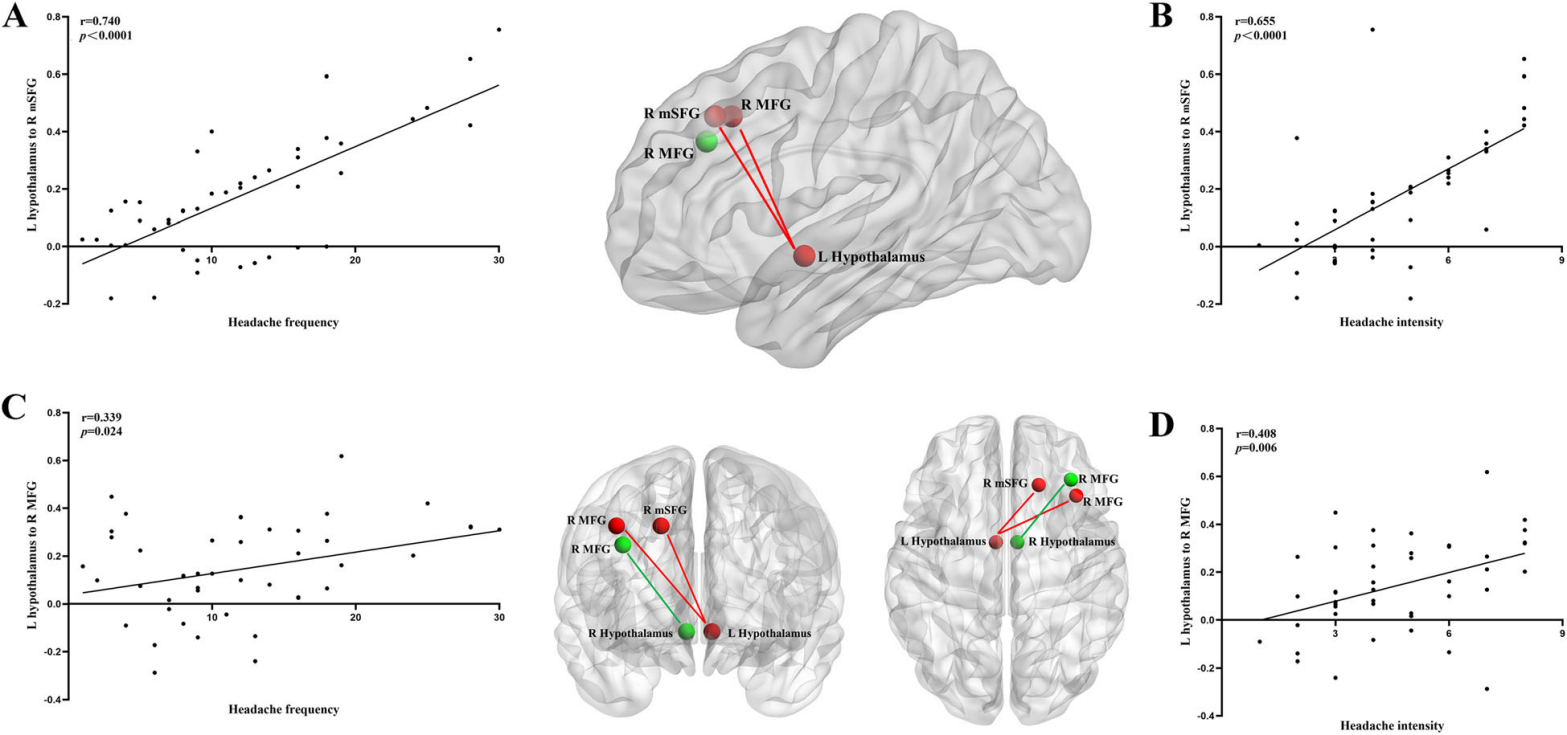
In mTBI patients with PTH, significantly positive correlations between the decreased functional connectivity of the left hypothalamus-right medial superior frontal gyrus and headache frequency (**a**) and headache intensity (**b**); and, significantly positive correlations between the functional connectivity of the left hypothalamus-right middle frontal gyrus and headache frequency (**c**) and headache intensity (**d**). The left hypothalamus, right hypothalamus, right medial superior frontal gyrus, and right middle frontal gyrus were visualized with the Brain Net viewer. L, left; R, right; MFG, middle frontal gyrus; mSFG, medial superior frontal gyrus

In the mTBI + PTH group at the acute stage, headache frequency was significantly positively correlated with the hypoconnectivity in the right mSFG and right MFG (*r* = 0.740, *p* < 0.001; *r* = 0.339, *p* = 0.024, respectively) (Fig. 3a and c). Headache intensity was significantly positively correlated with decreased FC between the right mSFG and right MFG (*r* = 0.655, *p* < 0.0001; *r* = 0.408, *p* = 0.006, respectively) (Fig. 3b and d). There were no significant correlations between headache symptom measurements and the FC of other identified regions.

## Discussion

mTBI can cause injury to the hypothalamus through damage to hypothalamic cell bodies. One study demonstrated both microstructural and functional connectivity abnormalities of the hypothalamus in mTBI patients by using diffusional kurtosis imaging (DKI) and resting state fMRI (rs-fMRI) [17]. The main finding in the present study was the significantly decreased FC between the hypothalamus and frontal gyrus in patients with mTBI with PTH compared with patients with mTBI without PTH. These decreased FC alterations were significantly correlated with the headache frequency and headache intensity. Frontal regions as part of the brain network are involved in pain processing including the affective and cognitive evaluation of pain. Notably, areas within the frontal lobe have previously been found to have abnormal structure, function, and FC in individuals with various pain disorders including PTH, migraine, cluster headache, and medication overuse headache [5, 18–20]. Several other studies have identified relationships between headaches and structural and functional brain alterations in the frontal gyrus [2].

Of interest, we found that the FC between the left hypothalamus and right MFG and that between the right hypothalamus and right MFG were significantly decreased in patients with mTBI with PTH compared with patients with mTBI without PTH. This reduced FC between the left and right hypothalamus and right MFG was significantly positively correlated with headache frequency and headache intensity, respectively. The MFG, as part of the prefrontal cortex that regulates pain perception [21], has been reportedly involved in both dorsal and ventral attentional networks, responses to fearful expressions, emotional management and the detection of salient behaviorally relevant but task-irrelevant stimuli in patients with migraine compared with controls [22]. In particular, the MFG is considered to play an important role in the cognitive evaluation and modulation of pain. Both abnormal neurostructural and functional alterations in the MFG have been reported in patients with PTH relative to healthy controls in previous studies [2, 23]. Structurally, patients with PTH showed decreased structural integrity (smaller surface area, volume or thickness, or higher curvature) in the MFG. In addition, one recent voxel-based morphometric (VBM) study showed that the decreased volume in the left MFG is significantly correlated with disease duration [24–26]. Functionally, a study by Dumkrieger and colleagues examined static and dynamic FC of 59 regions of interest involved in pain processing and found dynamic FC alterations in the left and right MFG in patients with PTH [12]. From this perspective, our results were consistent with those of researchers [27, 28]. The reason might be explained by the fact that impaired FC related to the MFG might diminish the capacity for pain evaluation and modulation.

We also demonstrated the significantly reduced FC between the left hypothalamus and right mSFG, and this decreased FC was significantly positively correlated with the headache frequency and headache intensity. The SFG region belongs to Brodmann area 10 (BA 10), which is the largest frontal brain region and is known as the anterior prefrontal cortex, frontopolar prefrontal cortex, or rostral prefrontal cortex. The cytoarchitecture of BA10 is characterized by more dendritic spines, a higher spine density, and a lower cell body density compared with other areas of the cortex, suggesting that BA 10 is more likely to accomplish these tasks of information integration [29]. In fact, BA 10 has been implicated in multiple integrative roles, including working memory, abstract reasoning, decision making, behavior control, nociceptive processing, and perceptual metacognition. Thus, the SFG, as one of the rich-club regions of BA 10, may have been involved in a great number of connections may lead to the modulation of cortical and subcortical nociceptive pathways. Compared with healthy controls, patients with PTH had less cortical thickness in the left and right SFG and demonstrated a negative correlation between left and right SFG thickness and headache frequency [2]. In addition, patients with pain disorders showed significant functional alterations, such as lower regional homogeneity (ReHo) and FC abnormalities in the superior frontal gyrus [24, 30]. More specifically, the medial frontal gyrus is known as the pain anticipation and modulation area through the anterior cingulate cortex (ACC), a key region of the pain matrix [30]. Sarmento et al. reported reduced N-acetylaspartate (NAA)/creatinine and increased choline/creatinine in the medial frontal lobe in patients with PTH compared with controls [31]. Our data of decreased FC between the left hypothalamus and right mSFG in mTBI patients with PTH are consistent with previous findings [32]. In the literature, this reduced FC may result in a permissive brain condition and the initiation of headache attacks, ultimately reflecting an insufficient pain modulating capacity.

Interestingly, our study demonstrated significant FC reduction between the left hypothalamus and contralateral MFG and mSFG. This asymmetry in connectivity during the resting state is small but consistent [33]. In the present study, hemispheric dominance may potentially play a role in defining the areas injured.

In the current study, we further showed that decreased FC between the left hypothalamus and left fusiform gyrus, left Rolandic operculum, right MFG and right mSFG as well as decreased FC between the right hypothalamus and right MFG, right postcentral gyrus and left supplementary motor area in mTBI patients compared with healthy controls. Among these brain regions, the fusiform is involved in nociception/antinociception and neurocognitive aspects of pain processing [34, 35]. In PTH, functional connectivity altered in the fusiform gyrus [12]. Evidence suggests that increased Rolandic operculum activation is correlated with some anticipatory processes. However, the exact role of the Rolandic operculum in pain disorders is unclear. fMRI studies have revealed activation of postcentral regions in response to painful stimulation [36]. Although no structural or functional alterations in the postcentral gyrus were found in PTH studies, cortical thinning, decreased amplitude of low-frequency fluctuation, hypoperfusion and FC abnormalities were shown in the postcentral gyrus in migraineurs [28]. The supplementary motor area, a region involved in motor planning and preparation, is activated in response to painful stimuli. Decreased supplementary motor area activation in patients with mTBI with PTH might indicate patients’ response to avoid movement during pain because physical movement worsens the pain [37, 38]. Nevertheless, these four brain regions were not significantly related to the hypothalamus in patients with mTBI with PTH. This may be attributed to the small samples of participants and requires further work to investigate why these brain areas are not involved in PTH.

Our study has several limitations. First, our research has a cross-sectional design with a small sample size. Second, the different parts of the hypothalamus have distinct roles; the anterior part seems to play an important role in headache attack generation and headache chronification, while the posterior part of the hypothalamus seems to be important for the acute headache stage. However, the entire hypothalamus, rather than hypothalamus subregions, was examined in this study. The current study could be extended to the hypothalamic subdivisions to investigate the FC patterns of mTBI patients with PTH. Third, scales to measure neuropsychological state, including depression and anxiety during a headache, were not measured; hence, the influence of these neuropsychological factors on hypothalamic FC was not assessed. Finally, the phenotype of PTH was not classified in detail, and different phenotypes may influence hypothalamic FC.

## Conclusion

In summary, this study revealed hypothalamic functional disconnections in mTBI patients with PTH compared to mTBI patients without PTH and healthy controls. There were associations between the reduced FC of hypothalamic-related brain regions and headache frequency and headache intensity. These results together could provide a new perspective to understanding the neuropathological mechanism underlying the PTH following acute mTBI to determine more appropriate management.

## Data Availability

Clinical, neuroimaging and statistical data will be available upon request from any qualified investigator.

## Abbreviations

mTBI: Mild traumatic brain injury
PTH: Post traumatic headache
ICHD-3: International Classification of Headache Disorder Third Edition
CT: Computed tomography
fMRI: Functional magnetic resonance imaging
FC: Functional connectivity
GCS: Glasgow Coma Score
VAS: Visual Analog Scale
MoCA: Montreal Cognitive Assessment
MNI: Montreal Neurological Institute
MFG: Middle frontal gyrus
mSFG: Medial superior frontal gyrus
VBM: Voxel-based morphometric
GMV: Gray matter volume
FOV: Field of view
FLAIR: Fluid-attenuated inversion recovery
SWI: Susceptibility weighted imaging
GRE: Gradient echo
